# Mirror image phosphoinositides regulate autophagy

**DOI:** 10.1080/23723556.2015.1019974

**Published:** 2015-02-26

**Authors:** Mariella Vicinanza, David C Rubinsztein

**Affiliations:** Department of Medical Genetics; Cambridge Institute for Medical Research; Cambridge, UK

**Keywords:** autophagym, phosphoinositide, VPS34, PI(5)P

## Abstract

Autophagosome formation is stimulated by canonical VPS34-dependent formation of phosphatidylinositol 3-phosphate [PI(3)P], which recruits effectors such as WIPI2. However, non-canonical VPS34-independent autophagy has also been proposed. We recently described that PI(5)P regulates autophagosome biogenesis, recruits WIPI2, and rescues autophagy in VPS34-inactivated cells. These alternative autophagy-initiating pathways reveal new druggable targets for treating neurodegeneration and cancer.

Phosphoinositides (PIs) are formed by phosphorylation of the inositol headgroup of phosphatidylinositol (**Fig. 1A**). The combined activities of multiple phosphoinositide kinases and phosphatases enable interconvertibility and rapid local changes in PI concentrations. When clustered, PIs create a cytosol-facing platform that enables the binding of machineries that regulate membrane deformation, and PIs can also influence the geometry of the membrane due to their inverted cone shape. Since local PI concentrations respond to nutrient availability and cell stress they can integrate membrane trafficking events within other functional modules, such as signaling and energy control.

**Figure 1.**
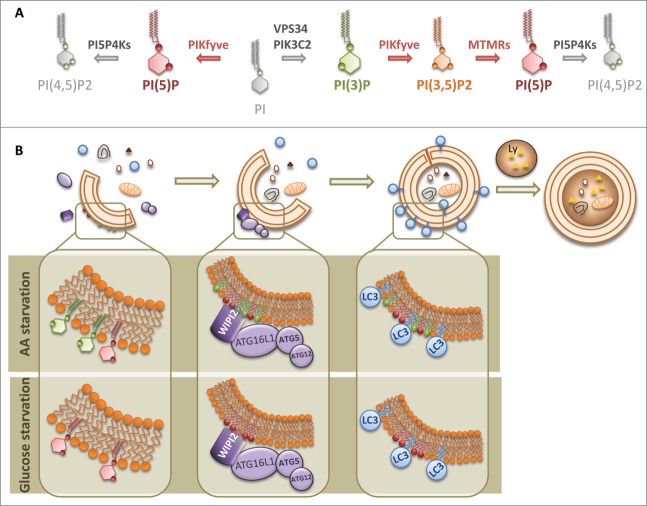
PI(5)P regulates autophagosome biogenesis. (**A**) Schematic representation of pathways for PI(5)P synthesis/turnover and enzymes involved in these pathways. PI5P4K, type II phosphatidylinositol 5-phosphate 4-kinase; PIKfyve, type III phosphatidylinositol 5-phosphate 5-kinase; VPS34, class III phosphatidylinositol 3-kinase; PIK3C2A, class II phosphatidylinositol 3-kinase; MTMR, myotubularin related 3-phosphatase. (**B**) PI(5)P (red) is found on pre-phagophore structures, phagophores, and autophagosomes, and stabilizes membrane-bound WIPI2 on autophagic precursors. WIPI2 binding recruits the ATG16L1-5-12 complex, which enables phagophore biogenesis and subsequent LC3 lipidation and autophagic substrate degradation. PI(5)P metabolism on autophagosomes seems to be highly dependent on PIKfyve and PI5P4K activities. Importantly, although we found that PI(3)P (green) was required for amino acid starvation-induced autophagy, PI(5)P synthesis was essential for autophagy activation by both amino acid and glucose depletion. Thus, we have identified PI(5)P as a new master regulator of glucose starvation-induced autophagy through a PI(3)P-independent autophagy pathway.

Autophagy is a bulk degradation process that delivers cytoplasmic materials (long-lived proteins and damaged organelles) to lysosomes, and can be induced by amino acid starvation, growth factor withdrawal, and low cellular energy levels. The first morphologically recognizable precursor of this process is a flattened double-membraned sac-like structure called a phagophore, whose edges elongate and fuse while engulfing a small portion of the cytoplasm to become an autophagosome (**Fig. 1B**). Autophagosomes fuse with lysosomes to form autolysosomes, whose contents can be degraded by the lysosomal hydrolases. Autophagy impacts the pathogenesis of diverse diseases, including neurodegenerative conditions, cancers, and infectious diseases.^1^

Autophagy has been classically considered to be dependent on phosphatidylinositol 3-phosphate [PI(3)P], since genetic studies in yeast and flies suggested that PI(3)P production by the class III phosphatidylinositol 3-kinase (VPS34) is required for autophagosome formation, for several proteins associated with autophagosome biogenesis (double FYVE-containing protein 1 [DFCP1] and WD-repeat protein interacting with phosphoinositide [WIPI1-2]),^2^ and for other components of the pathway.^3^ However, recent studies have also suggested the existence of VPS34-independent autophagy.^4^For instance, autophagosomes form in T lymphocytes and sensory neurons from *Vps34*^−/−^ mice^5^ and in glucose-starved or resveratrol-treated cells incubated with the VPS34 inhibitor wortmannin.^4, 6^ These phenomena may be attributed to VPS34-independent source(s) of PI(3)P,^7^ or to other phosphoinositides with similar properties to PI(3)P in the autophagy context.

The structure of PI(5)P is related to that of PI(3)P by rotation of the inositol ring that interconverts the position of the 3- and 5-phosphoryl groups, such that their charges can occupy equivalent positions (**Fig. 1A**). Although PI(5)P has been implicated in growth factor signaling pathways, chromatin organization, bacterial invasion, and cytoskeletal remodeling,^8^ our study is the first report of PI(5)P as an autophagy regulator.^9^ This is compatible with previous observations of similar binding of PI(3)P and PI(5)P to WIPI proteins.^10^

PI(5)P biosynthesis is regulated by the type III PtdInsP 5-kinase PIKfyve, either by direct phosphorylation of PI or via formation of PI(3,5)P2, which is then transformed into PI(5)P by 3-phosphatases of the myotubularin family (MTMRs)^8^ (**Fig. 1A**). The major route for PI(5)P removal is attributed to type II PI5PK kinases (phosphatidylinositol 5-phosphate 4-kinases, PI5P4K)^8^ (**Fig. 1A**). We found that increasing cellular PI(5)P levels (by the addition of exogenous PI(5)P or silencing of the PI5P4-kinases) enhanced autophagosome formation and autophagosome and autolysosome numbers, whereas depleting PI(5)P (via the PIKfyve inhibitor YM-201636 or by silencing of PIKfyve or MTMR3) decreased the numbers of autophagosome precursors and completed autophagosomes. Interestingly, PI(5)P (and its precursor PI(3,5)P2) localized on nascent and completed autophagosomes and the kinases acting on PI(5)P localized on autophagosomes (**Fig. 1B**) under different type of starvation conditions.

PI(5)P resembles PI(3)P as a regulator of autophagosome biogenesis. Acute depletion of PI(3)P using wortmannin completely ablated the presence of WIPI2 and DFCP1 vesicles and inhibited ATG5-12 conjugation and autophagosome formation. PI(5)P regulates autophagy even when VPS34 is inhibited, since the impaired autophagosome formation caused by wortmannin or VPS34 silencing was rescued by elevating PI(5)P levels. Our observations that PI(5)P binds to WIPI2, affects PI(3)P-related phenotypes such as ATG12-5 conjugation, and can rescue autophagy in wortmannin-treated HBSS-starved cells, argues that it can serve as an “alternative” to PI(3)P. Indeed, we found that PI(3)P and PI(5)P can mutually compete for binding to the PI(3)P autophagy effector WIPI2 *in vitro*. We propose that PI(5)P might account for the previously mysterious phenomenon of PI(3)P-independent, non-canonical autophagy. Since PI(5)P could sustain autophagy in VPS34-inactive cells, we further confirmed that PI(5)P was involved in glucose starvation- or resveratrol-induced autophagy, where PI(3)P is dispensable. Indeed, glucose starvation-induced autophagy appeared to be dependent on PI(5)P and not PI(3)P.

Our analysis reveals a hitherto unknown functional interplay between PIKfyve and PI5P4Ks in controlling PI(5)P levels in the context of autophagy and will pave the way for further studies of this exciting lipid in physiological cellular processes, including neurodegeneration and cancer. This may have therapeutic potential, because PIKfyve and PI5P4K enzymes are druggable targets and our data indicate that suppression of PI5P4K activity increased the clearance of autophagic substrates associated with neurodegenerative disease. Autophagy induction via other signaling effectors has benefits in a wide range of cell and animal models of neurodegenerative diseases caused by aggregate-prone intracytoplasmic proteins, like Huntington's and Parkinson's disease. Thus, PI5P4K inhibition may provide a tractable therapeutic target for such conditions. On the other hand, understanding the enzymes that regulate autophagy via PI(5)P may also enable inhibition of the pathway in cancers. This may be relevant in metastatic cancers where autophagy upregulation induced by the Warburg effect might contribute to tumor survival.
